# Therapeutic indications and other use-case-driven updates in the drug ontology: anti-malarials, anti-hypertensives, opioid analgesics, and a large term request

**DOI:** 10.1186/s13326-017-0121-5

**Published:** 2017-03-03

**Authors:** William R. Hogan, Josh Hanna, Amanda Hicks, Samira Amirova, Baxter Bramblett, Matthew Diller, Rodel Enderez, Timothy Modzelewski, Mirela Vasconcelos, Chris Delcher

**Affiliations:** Department of Health Outcomes and Policy, University of Florida, Clinical and Translational Research Building, 2004 Mowry Road, P.O. Box 100219, Gainesville, FL 32610 USA

**Keywords:** Biomedical ontology, Drug product, Therapeutic indication, Mechanism of action, Patient centered outcomes research, Anti-hypertensive, Anti-malarial, Opioid analgesic, Function, Disposition

## Abstract

**Background:**

The Drug Ontology (DrOn) is an OWL2-based representation of drug products and their ingredients, mechanisms of action, strengths, and dose forms. We originally created DrOn for use cases in comparative effectiveness research, primarily to identify historically complete sets of United States National Drug Codes (NDCs) that represent packaged drug products, by the ingredient(s), mechanism(s) of action, and so on contained in those products. Although we had designed DrOn from the outset to carefully distinguish those entities that have a therapeutic indication from those entities that have a molecular mechanism of action, we had not previously represented in DrOn any particular therapeutic indication.

**Results:**

In this work, we add therapeutic indications for three research use cases: resistant hypertension, malaria, and opioid abuse research. We also added mechanisms of action for opioid analgesics and added 108 classes representing drug products in response to a large term request from the Program for Resistance, Immunology, Surveillance and Modeling of Malaria in Uganda (PRISM) project. The net result is a new version of DrOn, current to May 2016, that represents three major therapeutic classes of drugs and six new mechanisms of action.

**Conclusions:**

A therapeutic indication of a drug product is represented as a therapeutic function in DrOn. Adverse effects of drug products, as well as other therapeutic uses for which the drug product was not designed are dispositions. Our work provides a framework for representing additional therapeutic indications, adverse effects, and uses of drug products beyond their design. Our work also validated our past modeling decisions for specific types of mechanisms of action, namely effects mediated via receptor and/or enzyme binding. DrOn is available at: http://purl.obolibrary.org/obo/dron.owl. A smaller version without NDCs is available at: http://purl.obolibrary.org/obo/dron/dron-lite.owl

## Background

The Drug Ontology (DrOn) is a Web Ontology Language version 2 (OWL2) based representation of drug products and their ingredients, mechanisms of action, strengths, and dose forms, as well as packaged drug products as represented by United States National Drug Codes (NDCs) [1–3]. The primary goal of DrOn is to support analyses of large, drug-related datasets such as pharmacy claims and electronic health record (EHR) data. Pharmacy claims datasets have traditionally been available to researchers from public and private payers (or third-parties that serve as the gateway to those payers’ data). In addition, state-wide prescription drug monitoring programs to combat opioid abuse are increasingly objects of study and capture NDCs of packaged drug products. These datasets use NDCs to identify the specific drug product that was dispensed to the patient as well as the drug product’s packaging and manufacturer. With the advent of research consortia and networks such as the Observational Health Data Sciences (OHDSI) collaborative [4] and the National Patient Centered Clinical Research Network (PCORnet) [5], massive EHR data sets with prescribing records normalized to the RxNorm terminology are increasingly available to researchers. RxNorm is a standard medication terminology built by the National Library of Medicine to standardize prescribing data [6].

Research using these increasingly available and growing claims and EHR datasets is facilitated by the ability to query their drug data based on various *characteristics* of the drug product prescribed or dispensed, such as therapeutic indication (e.g. hypertension) or mechanism of action of an active ingredient (e.g. beta blocker), rather than on the drug product itself. But the prototypical prescribing record in an EHR dataset identifies the drug product at the level of ingredient, dosage, and dose form—for example, furosemide 20 mg oral tablet—using a concept unique identifier from RxNorm (i.e., RxCui). The prototypical dispensing record in a claims database and prescription drug monitoring database uses an NDC to identify the drug product and its manufacturer and packaging: for example, a bottle of 100 furosemide 20 mg oral tablets from Sanofi-Aventis branded as Lasix. In the former case, the prescribing record will typically have an RxCui of 310429 for the furosemide 20 mg tablet. In the latter case, the dispensing record will typically have an NDC of 00039006710 for a bottle of 100 tablets. To query prescribing and/or dispensing records for all anti-hypertensives—or for all products with a beta-blocker as an ingredient—the researcher needs an easy way to generate lists of all RxCuis and/or NDCs, respectively, that represent drug products with these characteristics. *Our goal in creating DrOn was to create this ability*.

Artifacts that pre-existed DrOn were not sufficient for our purposes for various reasons. For example, any given version of RxNorm only contains the NDCs that were active at the time the version was released, but DrOn contains a historical record of NDCs. RxNorm, therefore cannot support querying historical EHR and claims datasets spanning multiple years and sometimes even decades without significant processing of past versions. This is not to criticize RxNorm: its primary use case was to support prescribing and ordering of medications in a clinical setting (and hence its central focus is the so-called Semantic Clinical Drug). In addition, RxNorm is not available as an OWL2 artifact to support integration with other ontologies such as those available from the Open Biomedical and Biological Ontology Foundry [7]. Unlike RxNorm and all other OWL2 artifacts of which we are aware, DrOn also represents the binding of ingredient compounds to cytochrome P450 (CYP) isoenzymes as either substrate, inhibitor, or both to support research on potential drug-drug interactions [1]. Lastly, although the National Drug File Reference Terminology (NDF-RT) is another OWL2 artifact with drug information such as therapeutic indications and mechanisms of action, it (a) makes basic scientific mistakes such as saying that ophthalmic timolol may treat systemic hypertension and that oral vancomycin may treat bacterial endocarditis and (b) has not been updated in its OWL2 form since 2013.

The increasing applicability and relevance of DrOn was the motivation for the work described here. Specifically, there were three use-cases from three major research-focused projects that included representing anti-hypertensive, anti-malarial, and analgesic therapeutic indications of certain drug products.

Although we had designed DrOn from the outset to avoid the scientific mistakes about therapeutic uses of drugs made by NDF-RT and other artifacts, prior to this work DrOn did not represent any particular therapeutic indication(s) of drug products. Furthermore, the use case motivating analgesic indications also required representing the mechanisms of action of opioid analgesics and antagonists, which we describe here. Also, the project motivating inclusion of information about which drug products are anti-malarials submitted a request for a large number of terms that resulted in additional significant development of DrOn that we report here. Lastly, we illustrate for the first time a method for querying DrOn to generate sets of RxCuis and/or NDCs to meet the use cases driving DrOn development.

## Methods

We first present the three research projects and the use cases that they presented. Then we describe our methodology for addressing the use cases. Lastly, we discuss a software tool that we developed and used to query sets of RxCuis or NDCs from DrOn.

### Use cases

#### OneFlorida clinical data research network

The OneFlorida Clinical Research Consortium is a state-wide infrastructure in Florida for implementation science, comparative effectiveness research, and pragmatic clinical trials [8]. It applied for and was awarded Phase II funding as a Clinical Data Research Network (CDRN) in the National Patient Centered Clinical Research Network (PCORnet). As part of PCORnet, OneFlorida is required to develop a patient cohort around a common condition, for which it chose hypertension [8].

**Fig. 1:**
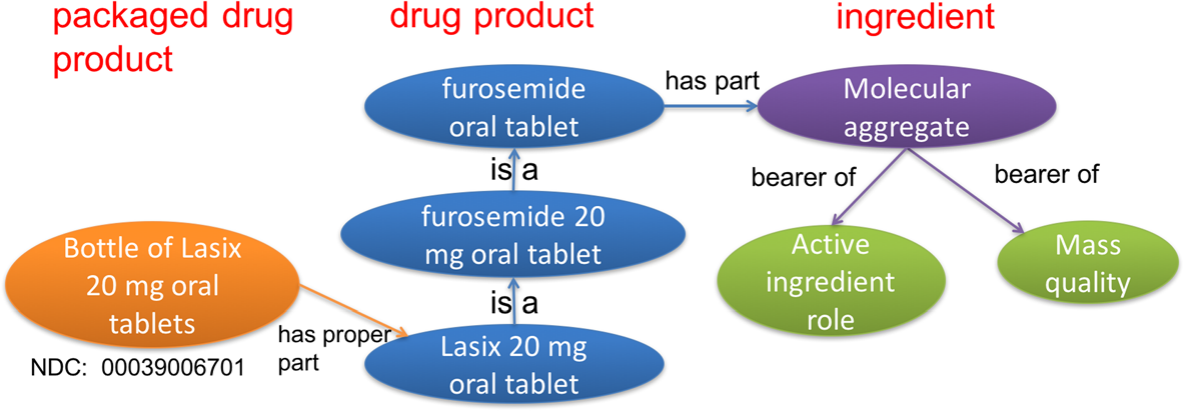
Representation of packaged drug products, drug products, ingredients, and the relationships among them in DrOn

Computable phenotypes are commonly used to identify a subpopulation of interest [9]. In particular, researchers in OneFlorida are studying resistant hypertension. Development of the resistant hypertension cohort requires extremely accurate counting of how many anti-hypertensives a patient is taking. To support the development of accurate computable phenotypes for hypertension that, for example, do not categorize patients as having hypertension who are receiving ophthalmic timolol but no anti-hypertensive drug, it was necessary to represent hypertension in DrOn as a therapeutic indication of drug products rather than of molecular compounds. Because the OneFlorida data warehouse—called the OneFlorida Data Trust—incorporates both claims and EHR data, it is necessary to query for lists of both NDCs and RxCuis which are used to standardize EHR data to identify the drug product prescribed (at the prescription stage, which manufacturer and packaging are not known or even typically specified). In DrOn, a drug product is a tablet, capsule, portion of solution, portion of cream, etc. (prescription) whereas a packaged drug product is a bottle of tablets or capsules, a tube of cream, a vial of solution, etc. for sale, distribution, delivery to the hospital ward from the pharmacy, etc. (dispensing). Figure 1 shows the relationships among packaged drug products, drug products, and ingredients as captured in DrOn.

#### Program for Resistance, Immunology, Surveillance and Modeling of Malaria (PRISM)

The Program for Resistance, Immunology, Surveillance and Modeling of Malaria in Uganda (PRISM) is an International Center of Excellence for Malaria Research (ICEMR) that serves East Africa. It is a collaboration between Makerere University in Uganda and the University of California San Francisco. The National Institute for Allergy and Infectious Disease (NIAID) created the ICEMR program in 2010 to establish a worldwide network of research centers in malaria-endemic settings to develop infrastructure for researchers and practitioners working in various settings, especially governments and healthcare institutions, to combat malaria.

PRISM required representing anti-malarial indications of drug products. It also submitted a request for a large number of terms to support matching drug codes in various data sets to DrOn. Upon cursory review, it appeared that many of these requests matched classes already in DrOn. However, after discussion it became apparent that PRISM required semantics for their requests that differed from the classes that we created in DrOn to match RxNorm. Specifically, RxNorm semantic clinical drugs (e.g. amoxicillin 50 mg/mL oral suspension) and semantic clinical drug forms (e.g. amoxicillin oral suspension) list all active ingredients exhaustively in a drug product—that is, they preclude the possibility of having additional active ingredients. For example, the class *amoxicillin oral suspension* does not subsume the class *amoxicillin/clavulanate oral suspension*, because it cannot have (by definition) additional active ingredients besides amoxicillin. The consequence is that in RxNorm (and therefore in DrOn) *amoxicillin oral suspension* is a sibling—not a parent—of *amoxicillin/clavulanate oral suspension*. Other combination drug suspensions with amoxicillin are also siblings. In addition, there is no common parent in RxNorm or DrOn for these drug products (i.e., there is no class *amoxicillin-containing suspension* as a common parent to the numerous siblings). However, PRISM required classes that had the semantics of *amoxicillin-containing suspension* that subsumes all kinds of suspensions containing amoxicillin (both with and without other active ingredients).

#### Prescription drug monitoring program research

Prescription Drug Monitoring Programs (PDMPs) such as Florida’s Electronic-Florida Online Reporting of Controlled Substance Evaluation Program (E-FORCSE®) [10] utilize databases of prescribed, controlled medications that include limited patient information, prescriber information, and NDCs for the dispensed medication. These databases may be accessed by prescribers (to view their prescribing histories), pharmacists, and law enforcement. Health researchers may also utilize this information to monitor (1) the introduction of newly controlled substances on the market, (2) medical morphine exposure in high-risk patient populations, (3) multi-drug prescribing associated with overdose (e.g. overlapping prescriptions of opioids and benzodiazepines), and (4) the impact of opioid prescribing policies (e.g. on reducing prescribing of short-acting formulations as the first option) on real-world prescribing behavior.

Opioid research with the PDMPs required the ability to query opioid analgesics based on (1) a combination of therapeutic indication (pain, medication assisted therapy for opioid dependence) and mechanism of action (binding to opioid receptors), (2) different mechanisms of action, specifically binding to mu, delta, and/or kappa opioid receptors in either antagonistic or synergistic ways, and (3) whether a drug is short- or long-acting. These queries are important to pharmacoepidemiologists for understanding the abuse potential and addictive properties of drug products.

### Methodology of DrOn development

Hogan et al. [3] and Hanna et al. [1, 2] describe our methods of developing DrOn. DrOn development occurs in two major parallel processes. The first process is traditional, manual editing using the Protégé ontology editor. We add to DrOn all information about the therapeutic indications and mechanisms of action of drug products and their ingredients through this manual process: we do not automatically import it from any other source. As described below, we search numerous sources of information to develop a comprehensive list of drug products with a particular indication or ingredients with a particular mechanism of action, and we then curate the information manually using Protégé. The second process involved in building DrOn is automated construction of classes from RxNorm.

**Fig. 2:**
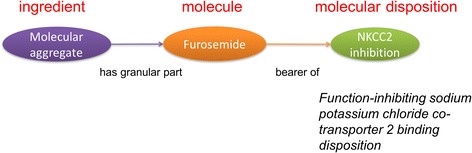
Representation of molecular dispositions to capture mechanisms of action in DrOn

DrOn is an OBO ontology and follows the OBO Foundry’s realist methods and principles of ontology development [7]. The ability to avoid confusing ophthalmic timolol as an anti-hypertensive and oral vancomycin as having any efficacy whatsoever against bacterial endocarditis was a key validation of the realist approach we took in [3]. This ability was the result of a key insight from our realist analysis that a therapeutic indication is a property of a drug product (tablet, ointment, cream, solution, etc.) with one or more active ingredients; whereas the mechanism of action is typically the property of a chemical compound (Fig. 2). For example, a molecule of timolol has the capability to competitively bind a beta-adrenergic receptor, but one molecule by itself has no ability to treat hypertension; it requires instead a number of timolol molecules on the order of Avogadro’s number to lower blood pressure. The drug product, therefore, has a sufficient number of molecules, as well as a formulation, that can be targeted towards a specific indication or indications. Thus, a timolol oral tablet drug product has the indication of hypertension (but not glaucoma); a portion of timolol ophthalmic solution has the indication of glaucoma (but not hypertension); and one timolol molecule by itself has no indication.

Besides dose form and intended route(s) of administration, the strength (quantity of active ingredient(s)) of a drug product also affects its therapeutic indication. For example, finasteride is prescribed in 5 mg dosages to treat benign prostatic hyperplasia but in 1 mg dosages to treat androgenetic alopecia. This example further supports our claim that the drug product, not the molecule, is the bearer of a therapeutic indication.

DrOn is available as both the full ontology, including NDCs, and a version without NDCs that includes all the manually edited content as well as content derived from RxNorm and imported from the Chemical Entities of Biological Interest ontology (ChEBI), the Protein Ontology (PRO), and other ontologies. This “lite” version of DrOn is available at [11]. The reader who is interested in reproducing our results on reasoning (methods and results discussed below) will find this version most amenable to the task. We recommend using the Fact++ reasoner in Protégé version 5.1, and adjusting the Java heap size to 2GB or more (4GB is recommended).

#### Methods for representing therapeutic indications

We analyzed therapeutic indications according to the realist perspective that we have maintained throughout the development of DrOn. Based on Hogan et al. we had already determined that a therapeutic indication is a property of a drug product and thus is some subtype of specifically dependent continuant per Basic Formal Ontology (BFO) [3]. Our analysis in this work focused further on whether it is more specifically a quality, role, or disposition, and if the latter, whether it is more specifically a function.

#### Methods for representing opioid analgesic mechanisms of action

Opioid-acting compounds primarily act by binding μ (*mu*), κ (*kappa*), and δ (*delta*) opioid receptors (there are as many as 17 kinds of opioid receptors in total). These receptors are located in the cellular membranes of peripheral and central nervous system neurons. The major effect of this binding is to prevent the release of neurotransmitters at the presynaptic nerve terminal, although opioid receptors also exist at the postsynaptic neuron with inhibitory effects. The consequent reduction in neurotransmission is what causes analgesic effects as well as side effects (or sometimes even desired effects) such as decreased bowel motility leading to constipation.

Authors SA, BB, RE, TM, and MV looked for lists of compounds with opioid activity in various resources, each one focusing on a particular resource. The resources we searched included DrugBank, NDF-RT, BioPortal, Ontobee, ChEBI, Wikipedia, and Goodman and Gilman’s Manual of Pharmacology and Therapeutics [12], as well as general Internet searches using Google. Each person also determined whether each compound on the list is used in drug products for analgesic activity. Once each person had generated as complete a list as she or he could from her or his assigned resource, we deduplicated the lists to produce a master list [13].

Once we had obtained a master list, the same authors subsequently searched the same kinds of resources for whether each compound was a *mu* agonist/antagonist, *kappa* agonist/antagonist, and/or a *delta* agonist/antagonist. Note that some compounds have agonist activity at one receptor but antagonist activity at another receptor. For example, fentanyl is a *mu* agonist and *delta* antagonist, although in bulk its *mu* agonist effects dominate such that it produces analgesia.

#### Methods for creating new classes

Authors SA, BB, MD, RE, TM, and MV each created one subset of the requested OWL classes for the PRISM project such that the union of these subsets covered all the requests for which we could identify the active ingredients. These six “class creators” created about 20 classes each. To avoid conflicts in the DrOn git repository on BitBucket, author JH created a fork of the repository called dron-workspace. Authors WRH and AH then set up a separate OWL file for each “class creator”. This allowed each one to check in his or her OWL file without any need for a merger that could break the RDF/XML in any OWL file. If another “class creator” had checked in his or her file before, synchronization was easily achieved by doing a “git pull” command before issuing a “git push” to the centralized repository. Finally, author JH merged the results of all six OWL files into a new dron-hand.owl module of DrOn.

For one group of term requests, route of administration was applicable. This group of terms involved drug solutions (i.e., one or more active ingredients dissolved in a liquid medium). Some drug solutions are designed for intravenous administration; some are designed for both ophthalmic and otic adminstration (i.e., one formulation can be administered either way); and some are formulated only for ophthalmic administration. We have not yet represented routes of administration of drug products in DrOn; this task is out of the scope of this paper and remains for future work. For drug solution terms, we created a generic “solution class” with an equivalent class definition. As an example, we define *gentamicin solution* as:

‘drug solution’ and (has_proper_part some (‘scattered molecular aggregate’

and (‘is bearer of’ some ‘active ingredient role’)

and (‘has granular part’ some gentamicin)))

Then we created classes gentamicin intravenous solution and gentamicin ophthalmic solution as primitive children of the gentamicin solution class.

### Software tool for querying DrOn

To query sets of RxCuis or NDCs from DrOn for use in research with EHR and claims datasets, we developed *dron-query*, an open-source, Java-based, command-line software application that uses the pre-existing OWL-API (Web Ontology Language Application Programming Interface) library. The *dron-query* application is available on GitHub at [14], and there is a getting started Wiki page at [15]. Essentially, dron-query (1) takes as input a description logic (DL) query formatted in Manchester Syntax (a standard syntax for writing description logic axioms in Protégé among others), (2) executes it using a description-logic reasoner, and (3) outputs for every class that meets the criteria specified in the query its (a) IRI, (b) rdfs:label, and (c) RxCui annotation value. If the query is for packaged drug products, the NDC is the rdfs:label output for the class, and the RxCui field in the output is null. But if the query is for drug products, the RxCui field is populated in addition to the IRI and rdfs:label.

We developed and executed DL queries using *dron-query* for the three use cases, and report the results.

## Results

We represented (1) anti-malarial function and asserted it as a function of 57 drug products (the reasoner infers it to be a function of an additional 203 drug products), (2) anti-hypertensive function and asserted it as a function of 326 drug products (inferred for 2419 additional products), (3) analgesic function and asserted it as a function of 413 drug products (inferred for 2779 additional products), and (4) six opioid mechanisms of action and asserted them as dispositions of 59 chemical compounds. We created 108 new classes in response to the PRISM term request.

### Therapeutic indications

A drug product has the potential to treat a disease or symptom or other condition, and this potential is only realized upon administration of the product to—and subsequent action on—an organism. Note that here we are using the word ‘potential’ in the sense of an ability or capability and not in the sense of probability of the ability or capability of being realized.

Administering a drug product does not guarantee realization of this potential; for example, one dose might be insufficient, or edema of the bowel wall might inhibit absorption of the drug, or the patient might have a genotype that results in excessive metabolism of the active ingredient into an inactive metabolite.

According to Basic Formal Ontology, this situation means that a therapeutic indication of a drug product is a realizable entity or one of its subtypes. BFO defines ‘realizable entity’ as *…a specifically dependent continuant that has at least one independent continuant entity as its bearer, and whose instances can be realized (manifested, actualized, executed) in associated processes of specific correlated types in which the bearer participates* [16].

Furthermore, because the physical makeup of the drug product confers this potential, and because physical changes to the drug product can cause it to lose its ability to treat instances of a particular type of symptom or disease, a therapeutic indication is a disposition (a subtype of realizable entity). BFO defines ‘disposition’ as *a realizable entity that is such that, if it ceases to exist, then its bearer is physically changed* [16]. Drug products can only lose their therapeutic potential through physical change. For example, a portion of epinephrine solution contained in a self-injection device degrades upon exposure to air and light, diminishing and ultimately removing with time its disposition to treat hypersensitivity reactions.

**Fig. 3:**
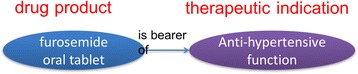
A drug product is the bearer of a therapeutic function

Lastly, for a therapeutic indication of a drug product for which the product was intentionally manufactured, a therapeutic indication is a function, which per BFO is a kind of disposition. BFO defines ‘function’ as *…disposition that exists in virtue of the bearer’s physical make-up, and this physical make-up is something the bearer possesses because of how it came into being—either through natural selection (in the case of biological entities) or through intentional design (in the case of artifacts)* [16]. Because drug products are extensively designed and planned to have certain therapeutic indications, these indications are functions. We also note that this usage is consistent with the most recent exposition of functions as they are represented by BFO [17]. Figure 3 illustrates the relationship of drug products to therapeutic functions.

Note that this means that given the definitions of ‘function’ and ‘disposition’ in BFO, the therapeutic potential(s) of a drug product for which the product was manufactured are functions, whereas therapeutic uses that are discovered later (for example, “off label” uses of a drug per the Food and Drug Administration) are initially dispositions that are not functions. Should the manufacturer subsequently manufacture the product with the intention of including these additional therapeutic indications, then these potentials are functions.

Note, however, that *no particular instance of disposition becomes an instance of function*. For example, consider a drug product that is a tablet with an indication (function) “to treat X.” Suppose that later researchers discover that the tablet also has the disposition “to treat Y.” Then all the tablets manufactured prior to the addition of this indication bear a function “to treat X” and a disposition “to treat Y”, whereas all the tablets manufactured after the change bear two functions: “to treat X” and “to treat Y.” It is possible that the exact same physical basis for the disposition exists for the function. This is analogous to the chopsticks example of Spear et al. where there are two identical sticks of wood (in terms of structure and form) one with the function “to handle food” because it was designed and manufactured to handle food, and one with a disposition but not a function “to handle food” because it came to have its structure incidentally [17].

Therefore, a therapeutic indication (function) is a subtype of therapeutic potential (disposition), where the former is the result of an intensively planned and executed manufacturing process and the latter is broader and includes effects of drug products that are discovered after they are manufactured. For example, bupropion tablets were originally designed and manufactured for depression. Later, researchers discovered that they also helped with smoking cessation, so companies started manufacturing these tablets for smoking cessation in addition to depression, ergo a new therapeutic function. Bupropion tablets manufactured prior to the change have one function; bupropion tablets manufactured after the change have two functions.

This distinction also helps to differentiate therapeutic indications from adverse effects of drug products. The realization of a disposition of an oxycodone tablet to cause constipation is an undesired effect. It is not a therapeutic indication (function). The disposition of oxycodone tablets to create a dependence (or addiction) is also an adverse event. We would represent in DrOn the dispositions to adverse events. Per the Ontology of Adverse Events (OAE), an adverse event is a process [18]. This process is typically the realization of certain dispositions of the drug product. Thus, we have also identified a key way to link DrOn to OAE: a disposition of a drug product (DrOn) is realized by an adverse event process (OAE).

In some cases, a type of disposition can have some instances whose realizations are therapeutic and other instances whose realizations are adverse events. For example, erythromycin when used to treat infection can have the adverse event of diarrhea caused by its disposition to increase gastric motility. However, the same disposition to increase gastric motility is sometimes used therapeutically to treat gastroparesis caused by diabetes mellitus. In other cases, the type of disposition can have some instances whose realizations are both therapeutic effects and adverse events, such as when the therapeutic effect is taken to an extreme (e.g., bleeding from anti-coagulant therapy and hypotension from anti-hypertensive therapy).

#### Anti-hypertensive therapeutic function

Def: a therapeutic function of a drug product that is realized by administration of the drug product resulting in a decrease of systemic arterial pressure.

Hypertension is a sustained elevation in the pressure exerted by blood on the systemic arteries (as opposed to pulmonary arteries) of an organism. This condition is well known to be a risk for multiple morbidities including coronary artery disease, stroke, and kidney disease. Drug products manufactured to treat hypertension all have the disposition of lowering this pressure when administered in the proper form and according to the proper route of administration.

#### Anti-malarial therapeutic function

Def: a therapeutic function of a drug product that is realized by administration of the drug product resulting in creation of a material basis of a resistance to malaria infection disposition.

In other words, a drug product like a choloroquine tablet confers upon administration a protective resistance to an infection of a certain kind, namely protective resistance to individuals of one of four Plasmodium species. The Infectious Disease Ontology defines ‘protective resistance’ as: *A disposition that inheres in a material entity in virtue of the fact that the entity has a part (*e.g. *a gene product), which itself has a disposition to mitigate damage to the entity* [19]. Resistance to an infectious agent then is a type of protective resistance. This kind of resistance can be either acquired or innate (e.g., an extreme but common case of innate resistance is the resistance of one species to the infectious agents that commonly infect another species). Acquired resistance can occur through acquired immunity, through administration of anti-infective drug products, and through other mechanisms.

An anti-malarial is thus a drug product that, when administered, confers a protective resistance to humans against the four species of Plasmodium that cause malaria in humans. DrOn imports ‘resistance to malaria infection’, which is itself defined as *A resistance to infection by P. vivax, P. ovale, P. malariae, and/or P. falciparum*.

#### Analgesic therapeutic function

Def: a therapeutic function of a drug product that is realized by administration of the drug product resulting in blocked realization of a disposition to pain.

We follow the definition of ‘pain’ by Smith and Ceusters as a type of process: *an unpleasant experience on the part of a human subject that is both sensory and emotional and that is of a type that is either canonical pain … or phenomenologically indistinguishable from canonical pain* [20]. The physical basis of the pain can range from activation of the nociceptive system (canonical pain) to damage to the nociceptive system (neuropathic pain) to changes in the cognitive system (e.g., pain behavior without nociception).

The net result is that certain physical changes result in a disposition to experience pain, and the ultimate effect of analgesics is to block the realization of this disposition. Restated, they confer a blocking disposition to pain dispositions.

### Adding mechanisms of action for opioid analgesics

We defined six dispositions, all of which inhere in molecules. There are two each (agonistic and antagonistic) for each of the three major opioid receptors that were relevant to the E-FORCSE use case. These definitions all follow the template:

A disposition of a molecule to bind an instance of < kind of receptor > in a manner that < activates, inhibits > the realization of the biological function of the receptor.

We then link the function to the molecule using OWL2 axioms of the form:

<molecule> subClassOf (‘is bearer of’ some <disposition>)

(where ‘is bearer of’ is an object property, <molecule > is a class, and < disposition > is a class).

Note that for compounds in DrOn that are represented by ChEBI class internationalized resource identifiers (IRIs), we are asserting this axiom directly on ChEBI classes (which is true of nearly all the opioid compounds). Furthermore, we note that we do not yet represent in DrOn the processes that realize these dispositions, nor the other participants in these processes (e.g., the *mu* receptor itself). The reason is that our use cases have not yet required it. However, we acknowledge that it might be useful to query for all drug products whose ingredients act on *mu* receptors, regardless of whether the action inhibits or activates the receptor. It therefore remains future work to enhance the representation in DrOn to include the processes that realize molecular dispositions and their participants. We note that the Gene Ontology [21] has terms for the processes (e.g., GO:0031698 *beta-2 adrenergic receptor* binding), and the Protein Ontology [22] has terms for the receptors (e.g., PR:000001193 *beta-2 adrenergic receptor*) that should be reused for this purpose. For more on DrOn’s representation of drug ingredients (molecules and aggregates of them) and their dispositions and roles, see Hanna et al. [1].

All told, we added at least one of the six dispositions to 59 molecule types (Table 1).

**Table 1:** Counts of molecules with various opioid mechanisms of action

Action	Type of receptor		
	*mu*	*kappa*	*delta*
Agonist	28	11	9
Antagonist	4	3	3

We note that not all drug products with (an ingredient that has) an opioid agonist mechanism have an analgesic indication. For example, loperamide tablets are used to treat diarrhea and are not indicated for pain relief (because they bind opioid receptors in the nerve cells of the gut almost exclusively).

### PRISM term request

**Fig. 4:**
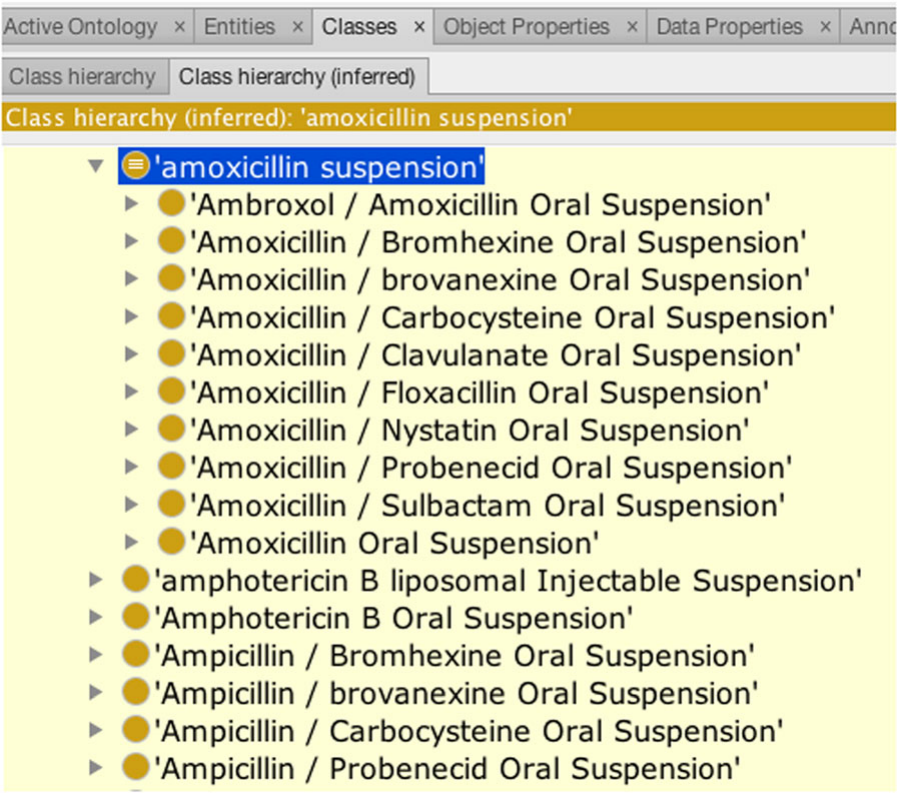
The new class *amoxicillin suspension* and its inferred children in DrOn

We added 108 classes in response to the PRISM term request: 89 classes have an equivalent class axiom, and 19 drug solution classes have necessary axioms only. Figure 4 shows the class *amoxicillin suspension* and that the Fact++ reasoner has inferred that the 10 amoxicillin suspension classes (that derive from RxNorm) are subsumed under it.

### Using dron-query tool for the use cases

#### Anti-hypertensive query

We used dron-query to query several lists of RxCuis for the anti-hypertensive use case. Specifically, we queried all the RxCuis for anti-hypertensive products whose ingredients had one of the following mechanisms of action, with one set of RxCuis per mechanism of action: beta-adrenergic blockade, calcium channel blockade, NKCC2 inhibition (loop diuretics), sodium-chloride co-transporter inhibition (thiazide and thiazide-like diuretics), angiotensin converting enzyme inhibition, and angiotensin receptor blockade.

The template for each query in Manchester syntax was the following:

'drug product' and ('is bearer of' some 'anti-hypertensive function') and (has_proper_part some ('has granular part' some ('is bearer of' some < *disposition to bind some enzyme/receptor>* )))

For beta blockers, the exact query is:

'drug product' and ('is bearer of' some 'anti-hypertensive function') and (has_proper_part some ('has granular part' some ('is bearer of' some 'non-activating competitive beta-adrenergic receptor binding disposition')))

This query returns 612 classes representing drug products at multiple levels of granularity (i.e., metoprolol oral tablet, metoprolol 50 mg oral tablet, Lopressor 50 mg oral tablet).

To get a set of NDCs for anti-hypertensive drug products with beta blocker ingredients, the query is:

‘packaged drug product’ and (has_proper_part some ('is bearer of' some 'anti-hypertensive function') and (has_proper_part some ('has granular part' some ('is bearer of' some 'non-activating competitive beta-adrenergic receptor binding disposition'))))

This query returns 4,831 classes and their NDCs.

#### Anti-malarial query

For all drug products with an anti-malarial function, the query is:

'drug product' and ('is bearer of' some ‘anti-malarial function’)

This query returns 260 drug products and their RxCuis.

#### Opioid analgesic query

For all analgesics that have a *mu* agonist as an ingredient, the query is:

'drug product' and ('is bearer of' some analgesic) and (has_proper_part some ('has granular part' some ('is bearer of' some 'mu agonist')))

This query returns 3034 drug products and their RxCuis. The analogous query for packaged drug products (not shown) returns 8142 packaged drug products and their NDCs.

## Discussion

Based on use cases from three research projects, we extended DrOn with three therapeutic functions and six mechanisms of action of drug products. We also added 108 classes in response to term requests, including amoxicillin suspension and chloroquine tablet. These classes have different semantics than RxNorm classes because they do not exhaustively list all active ingredients. For example, the new class amoxicillin suspension subsumes all suspensions that have amoxicillin as one ingredient, including compound drug products such as amoxicillin/clavulanate oral suspension. Lastly, we illustrated the use of the *dron-query* software tool for the three use cases.

The three therapeutic functions—anti-hypertensive, anti-malarial, and analgesic—are fairly diverse, providing preliminary evidence that our approach is general. There are no common biological pathways, or even organ systems, to hypertension, malaria, and pain. Although the blood is involved with both hypertension and malaria, the anti-hypertensive drugs all have a mechanism of action that take place elsewhere (especially the kidneys, where they bind cellular receptors).

We have laid an ontological basis for representing additional therapeutic indications, off-label usages, and adverse effects of drug products. And per the original design of DrOn, we capture the therapeutic indication on the appropriate entity (drug product and not molecule). In so doing, we do not mistake ophthalmic timolol for an anti-hypertensive or oral timolol for a glaucoma drug. Representing actual dispositions of drug products towards adverse events, and thereby linking DrOn and OAE, remains future work.

DrOn is extensible in the manner described for representation of additional therapeutic functions. Ongoing work at present includes representing the therapeutic function to treat asthma.

In addition, our past approach to representing mechanisms of action was in this work easily adapted to opiates and opioids. However, we note that all the mechanisms of action represented in DrOn to date involve drug molecules binding to molecular entities in the cellular membrane (e.g., receptors, enzymes, and transporters). Representing mechanisms of action that involve other kinds of processes might not follow this pattern. For example, because anti-infectives act on a symbiont of the organism to which they are administered, as opposed to the organism itself, representing them could be more complex. Nevertheless, the space of compounds that exert a biological effect through receptor binding and enzyme inhibition is large and diverse.

We do not at present relate the molecular mechanism(s) of action of a molecule to the relevant therapeutic functions of a drug product that incorporates the molecule, or vice versa. Thus, for atenolol oral tablet there is no connection between its function to treat hypertension and the dispositions of its atenolol molecules to bind beta-adrenergic receptors. This task is future work. Because our representation does not require capturing the relationship between mechanisms of action and therapeutic functions however, we can represent in DrOn the therapeutic functions of drug products for which no mechanism of action at the molecular level is yet known.

For the PDMP research, we have begun but not completed work on whether particular opiate and opioid-containing drug products are short vs. long acting formulations. We merely note here that duration of action is a property of not just the half-life of the active ingredient(s) but also the particular way a dose is manufactured (e.g., normal vs. delayed vs. extended release tablets and capsules) and the genotype of the individual (e.g., single nucleotide polymorphisms that result in poor vs. ultra-rapid metabolism of drugs and prodrugs).

This work was limited to the extensions necessary to meet specific research use cases. We have thus not studied clinical uses. However, we note that were a system such as an EHR to recommend oral vancomycin to treat endocarditis, or ophthalmic timolol to treat hypertension, a clinician might begin to doubt the system. In addition, for our research use cases, we have not yet compared counting anti-hypertensives, hypertensive patients, or patients with resistant hypertension using DrOn to counting them using NDF-RT or other artifacts. This task remains as future work.

We also did not represent the intended routes of administration of drug products, which prevented inclusion of an equivalent class axiom on all classes added in response to PRISM term requests. This task also remains future work. We note that the Vaccine Ontology has a set of classes representing routes of administration that will likely be applicable [23].

We also note that none of the use cases discussed here relate yet to pharmacogenomics or analyzing pharmacodynamics or pharmacokinetics of drugs. However DrOn is being used in other projects in this manner: we refer the interested reader to Brochhausen et al. [24], which discusses ontological representations of potential drug-drug interactions and their pharmacodynamics and pharmacokinetics, and how these representations reuse DrOn classes.

## Conclusions

We successfully captured therapeutic indications of drug products in the Drug Ontology as functions and other therapeutic uses of drugs as dispositions, in keeping with the definitions of ‘disposition’ and ‘function’ in Basic Formal Ontology. We represented the anti-hypertensive, anti-malarial, and analgesic indications of numerous drug products in DrOn in this manner. We also represented the mechanisms of action of opioid analgesics (and other opioid drug products), and we included over 100 new classes in response to a term request from the PRISM project. We also demonstrated how to use the *dron-query* tool to extract from DrOn subsets of drug-product and packaged-drug-product classes and their annotations for various use cases. To date, our results show promise that our methods are applicable to other therapeutic indications and mechanisms of action of drug products and their ingredients, respectively.

## Abbreviations

BFO: Basic formal ontology
CDRN: Clinical data research network
CYP: Cytochrome P450
DrOn: Drug ontology
E-FORCSE: Florida’s electronic-Florida online reporting of controlled substance evaluation program
EHR: Electronic health record
ICEMR: International center of excellence for malaria research
NDC: National drug code
NDF-RT: National drug file reference terminology
NIAID: National institute for allergy and infectious disease
OAE: Ontology of adverse events
OBO: Open biological and biomedical ontologies
OWL2: Web ontology language version 2
PCORnet: National patient-centered clinical research network
PDMP: Prescription drug monitoring program
PRISM: Program for resistance, immunology, surveillance and modeling of malaria in uganda
RDF: Resource description framework
XML: eXtensible markup language
